# What undermines healthy habits with regard to physical activity and food? Voices of adolescents in a disadvantaged community

**DOI:** 10.1080/17482631.2017.1333901

**Published:** 2017-06-06

**Authors:** Linus Jonsson, Christel Larsson, Christina Berg, Peter Korp, Eva-Carin Lindgren

**Affiliations:** ^a^ Department of Food and Nutrition, and Sport Science, University of Gothenburg, Gothenburg, Sweden; ^b^ School of Health and Welfare, Halmstad University, Halmstad, Sweden

**Keywords:** Barriers, child’s perspective, focus-group interviews, food habits, gender perspective, physical activity, youth

## Abstract

This study aimed to illuminate factors that undermine the healthy habits of adolescents from a multicultural community with low socioeconomic status (S.E.S.) in Sweden with regard to physical activity (P.A.) and food, as stated in their own voices. Adolescents (*n* = 53, 12–13 y/o) were recruited from one school situated in a multicultural community characterized by low S.E.S. Embracing an interpretive approach, 10 focus-group interviews were conducted to produce data for the study. The focus-group interviews were audio recorded, transcribed verbatim, and analysed using qualitative content analysis. The analysis resulted in two major themes: (1) the availability of temptations is large, and support from the surroundings is limited; and (2) norms and demands set the agenda. The adolescents’ voices illuminate a profound awareness and the magnitude of tempting screen-based activities as undermining their P.A. and healthy food habits. Moreover, several gender boundaries were highlighted as undermining girls’ P.A. and healthy food habits. The adolescents’ stories illuminated that it is difficult for them, within their environment, to establish healthy habits with regard to P.A. and food. To facilitate the adolescents’ healthy habits, we suggest that support from family, friends, the school, and society at large is essential.

## Introduction

Immigrants in Sweden and Europe, particularly those from non-EU countries, are severely disadvantaged socioeconomically when it comes to education, employment opportunities, and earnings (Dustmann & Frattini, 2012). Unfortunately, socioeconomic inequalities are also apparent in adolescents’ health, and over the past few years, these inequalities have increased (Elgar et al., 2015). Overall, adolescents from families with low socioeconomic status (S.E.S.) report poorer self-rated health and life satisfaction, are less likely to eat fruits and vegetables on a daily basis, and are less likely to achieve 60 min of moderate-to-vigorous physical activity (P.A.) a day compared to their more privileged counterparts (WHO, 2016).

Few children and adolescents in general meet the current recommendations concerning P.A. (Kalman et al., 2015) and dietary intake (Nordic Council of Ministers, 2017; Vereecken et al., 2015). In relation to this, adolescent girls tend to engage in less P.A. compared to their male counterparts, whereas adolescent boys report higher consumption of soft drinks and lower intake of fruits compared to girls (WHO, 2016). Thus, some studies have shown interest in adolescents’ perspectives and experiences of what influences their P.A. and dietary intake (e.g., Krølner et al., 2011; Martins, Marques, Sarmento, & Carreiro da Costa, 2015).

Scholars exploring adolescents’ perspectives have identified a number of factors that undermine healthy habits with regard to P.A. and food at an individual level, social level, environmental level, and societal level (Jenkins & Horner, 2005; Martins et al., 2015; Shepherd et al., 2005; Spencer, Rehman, & Kirk, 2015; Stevenson, Doherty, Barnett, Muldoon, & Trew, 2007). However, the majority of previous studies has been conducted in the US and the UK (e.g., Krølner et al., 2011; Martins et al., 2015; Shepherd et al., 2005; Spencer et al., 2015), and relatively few have focused on the voices of children and adolescents from multicultural communities with low S.E.S. (see, for example, McEvoy, MacPhail, & Enright, 2016; Rawlins, Baker, Maynard, & Harding, 2013; Taverno Ross & Francis, 2016; Tiedje et al., 2014). Unlike other studies, these studies have, for example, identified safety concerns in the neighbourhood and monetary cost as barriers to P.A. (Rawlins et al., 2013; Taverno Ross & Francis, 2016) and “Americanized eating practices” with assimilation to fast-food culture and cost as undermining healthy food habits (Rawlins et al., 2013; Tiedje et al., 2014). Similarly, there are gender-specific factors such as norms, ideals, and stereotypes that undermine girls’ P.A. and healthy food habits especially (Spencer et al., 2015).

As argued by Patton (2015), however, earlier findings must be understood within the context of the time and space in which they occur. Moreover, the differences in results generated from the voices of adolescents from different settings imply the importance of further studies of adolescents from multicultural communities with low S.E.S. Listening to the voices of adolescents from various and less advantageous settings also aligns with the United Nations Convention on the “Rights of the Child”, which emphasizes that every child under the age of 18 has the right to express his or her opinion and to be heard in matters affecting his or her health and well-being (United Nations Human Rights, 2016). As such, the purpose of this study is to illuminate factors that undermine the healthy habits of adolescents from a multicultural community with low S.E.S. in Sweden with regard to P.A. and food, as stated in their own voices. More specifically, this study aims to answer the following two research questions: (1) What do the adolescents express as undermining factors in relation to healthy habits with regard to P.A. and food? (2) How can the undermining factors expressed through the adolescents’ own voices be understood from a gender perspective?

### Perspectives

This paper embraces the perspective of children. In doing so, it considers both the children’s perspective and a child perspective (Thulin & Jonsson, 2014). Through the children’s perspective, this study intends to listen to the adolescents’ own voices (Thulin & Jonsson, 2014) concerning healthy habits with regard to P.A. and food. This is essential in order to gain insight and understanding of adolescents’ perspectives concerning P.A. and healthy food habits (cf. Spencer, 2014). Based on a child perspective, the adolescents’ own voices will be interpreted by us as researchers (Thulin & Jonsson, 2014) through a gender perspective in order to gain a deeper understanding of the adolescents’ perspectives. This is crucial since gender norms and ideals create dominant symbols and belief systems that influence social structures, institutions, practices, and relationships, as well as individual thinking and behaviour (Connell, 1987). Therefore, it is important in order to be able to critically reflect upon what kinds of support adolescents from multicultural areas with low S.E.S. might need in relation to healthy lifestyle habits.

The idea of “boundaries” has gained increasing attention in the last few decades across the social sciences (Lamont & Molnár, 2002). Figuratively speaking, boundaries are lines or borders that outline patterns of likes and dislikes, which are socially constructed (Lamont, 2001). Such boundaries are apparent in what individuals think and do and how organizations operate. It has been hypothesized that these boundaries are similar to membranes, meaning that they are malleable or subject to change (Lamont & Molnár, 2002). Further, Lamont and Molnár (2002) make a distinction between symbolic boundaries and social boundaries. On the one hand, symbolic boundaries represent conceptual distinctions that social actors employ to categorize objects, people, practices, and space and time. On the other hand, social boundaries signify more fixed and “objectified forms of social differences manifested in unequal access to and unequal distribution of resources (material and nonmaterial) and social opportunities” (Lamont & Molnár, 2002, p. 168). Social boundaries surface through symbolic boundaries and are displayed in tangible inequalities (Lamont & Molnár, 2002).

## Methodology

This study is based upon an interpretive approach, which entails that the constructed and contextual nature of the adolescents’ experience is complex, and it allows for shared realities (Thorne, 2008). In order to capture the adolescents’ complex and shared realities, this study relied on focus-group interviews to produce data, as this allowed the adolescents to express themselves freely and make their voices heard (Dahlin Ivanoff & Hultberg, 2006).

### Setting the scene

During 2015, more than one million people fled their homes and came to Europe because of persecution, conflicts, and poverty (United Nations Refugee Agency, 2016). Related to this, socioeconomic segregation in Europe, including Sweden, is increasing (Musterd, Marcińczak, van Ham, & Tammaru, 2015). Gothenburg is the second-largest city in Sweden, with about 550,000 residents. In recent years, socioeconomic segregation in Gothenburg has increased, and Angered is one of the most segregated districts of the city (Göteborgs Stad, 2014). Angered is characterized, according to Swedish standards, as having a low average income, a high proportion of people of foreign origin, long-term financial assistance, long-term unemployment, low voter turnout, low educational level, poor self-reported health, and poor life expectancy (Göteborgs Stad, 2014). In Angered, there is a school attended by approximately 450 students in grades four to nine. By Swedish standards, this school has received a large share of newly arrived students, and the number of students with a foreign background is well above average while the proportion of students in ninth grade who pass all subjects and educational achievement score is below average (see Table 1; The Swedish National Agency for Education, 2016).

**Table 1.** Descriptive data for the school at which the data production took place.

Variable	School at which the data production took place	National mean
Socioeconomic status^a^	1.66	2.26
Educational achievement score^b^	185	225
Pass in all grades (%)	45.2	77.0
New arrivals (%)^c^	19	5
Foreign background^d^	92	21

^a^The mean of the parents education level with 1 point for completed compulsory school; 2 points for completed upper secondary school; and 3 points for ≥20 credits from post-secondary education.

^b^The score of 16 subjects in the curriculum ranging between 0 and 320 points with 20 points for grade A, 17.5 for B, 15 for C, 12.5 for D, 10 for E, and 0 for F.

^c^Foreign-born pupils with foreign-born parents, arrived to Sweden during the last 4 years with no previous experience of the Swedish compulsory school system.

^d^Foreign-born pupils and pupils born in Sweden with both parents born outside Sweden.

### Recruitment and participants

During September and October 2014, all 54 seventh graders (ages 12–13 years) who attended the school described above were invited to participate in the present study. All agreed to take part; however, one adolescent was absent during his scheduled focus-group interview. This resulted in a total sample of 53 adolescents comprised of boys (*n *= 21) and girls (*n *= 32). The adolescents who comprised the sample for the present paper were enrolled in an ongoing research project called “How-to-Act?”, which is an empowerment-based health-promoting school intervention programme. The adolescents and their parents or legal guardians received written and oral information about the research project, including its purpose; they were informed that participation was voluntary and that the adolescents could terminate their participation at any time with no repercussions. The written information was also translated to Arabic and Somali. Informed consent was obtained from both the adolescents and their parents or legal guardians. The research project and this study have been approved by the regional ethical committee (Dnr: 469–14).

### Procedure and data production

At an initial stage, two focus-group interviews were conducted with adolescents of the same age but from another school as a way to pilot test the focus-group interviews. This pilot testing, in addition to inspiration from the literature (e.g., Dahlin Ivanoff & Hultberg, 2006), formed the basis for how the focus-group interviews in the present study were designed. In total (excluding the pilot interviews) 10 focus-group interviews were conducted with four to six adolescents in each. Four of the focus groups were with girls only, three with boys only, and three were mixed-gender focus groups. During each focus-group interview, three interviewers were present; two were Ph.D. students (with no prior experience of focus-groups interviews except from the pilot testing), and the third was one of three senior researchers (all with prior experience of focus-groups interviews). One of the two Ph.D. students always served as a main moderator with the primary responsibility of keeping the discussion going and focused. The other two interviewers both served as assistant moderators and, consequently, asked questions when suitable opportunities arose. An important aspect was to develop a relationship of trust between the interviewer and the adolescent, since focus group interviews can be significantly constrained by the power imbalance between the adolescents and the adults as well as between the adolescents (Green & Hogan, 2005). Also, in the beginning of each focus group interview, an explanation was given that all statements would remain within the group, there are no right or wrong answers, and all thoughts and opinions are important.

To guide the focus-group interview, a figure was placed on the table with the words “health”, “food”, “physical activity”, “body”, and “wishes”. To initiate the discussion, the main moderator pointed to the figure and described that these were the themes that was going to be discussed, and started by asking general questions such as “what is health?” and/or “what does health mean to you?”. During the focus-group interviews one of the assistant moderators summarized what the adolescents had expressed to ensure correct interpretation, and as a way to elicit elaboration on the topics. A similar procedure was used for the themes “food”, “physical activity”, and “body”. Each session ended with one of the assistant moderators summarizing the adolescents’ expressed “wishes” (e.g., wish for support, desire to change) in relation to each theme (i.e., health, food, P.A, body) on a whiteboard. About halfway into each focus group, the adolescents were offered refreshments (i.e., fruit and juice). The focus-group interviews lasted from 44 to 97 min (an average of 69 min).

### Data analysis

The primary (inductive) analysis of the focus-group interviews was done using qualitative content analysis (Graneheim & Lundman, 2004; Krippendorff, 2013). Qualitative content analysis was considered suitable for making an inductive thematization of the data to obtain a description of the undermining factors concerning P.A. and healthy food habits made visible through the adolescents’ voices. As such, the primary analysis relates to the children’s perspective (Thulin & Jonsson, 2014). Adhering to the child perspective (Thulin & Jonsson, 2014), the secondary (deductive) analysis (i.e., a theoretical analysis) was done using a gender perspective (Barker-Ruchti, Grahn, & Lindgren, 2016; Lamont & Molnár, 2002). The following four steps were performed in the qualitative content analysis.

1. At an initial stage, the interviews were transcribed verbatim, and the transcriptions were read through several times in order to “obtain a sense of the whole” (Graneheim & Lundman, 2004, p. 108) and to gain an overall understanding of the adolescents’ perspectives about undermining factors.
2. In order to go deeper into details and to identify similarities and differences in the empirical data, a back-and-forth process between decontextualization and contextualization was performed. Thereafter, the text was divided into meaning units, and then the meaning units were condensed. The condensed meaning units were then abstracted and coded (see Table 2). Similarities and differences between the codes were then sorted and compared to each other, and thereafter arranged into tentative categories. The tentative categories were reviewed and discussed several times, and then revised and encoded into final categories. Up to this stage, the analysis focused on the visible and obvious components of the transcriptions.
3. Next, we moved between the empirical data and the literature to identify adequate theories that would help us acquire a deeper understanding and to better interpret the adolescents’ perspectives.
4. In the last step, the empirical findings concerning the contents of the categories were compared and contrasted with a gender perspective. It was also in this step that the categories were arranged into the final themes.

**Table 2.** Examples of meaning units, condensed meaning units, codes, categories, and themes from the analytic procedure.

Meaning unit	Condensed meaning unit	Code	Category	Theme
- It’s not like I eat vegetables to comfort eat when I’m sad. Rather, I eat chips and stuff.	Comfort eating chips while sad.	Comfort eating.	Emotional eating.	The availability of temptations is large, and support from the surroundings is limited.
- Some do not want to; I don’t think girls should do sports because they cannot. If we play football, they cannot play football.	Girls should not play sports because they cannot.	Sports are not for girls.	Physical activity is mainly for boys.	Norms and demands set the agenda.

The analysis was performed by the first author; however, all steps were regularly discussed among the co-authors in order to reach a consensus. The analysis is described in a linear fashion; during the actual analysis, however, we worked back and forth with the codes, categories, and themes.

## Findings and discussion

The analysis resulted in two major themes that included eight categories: (1) *t* *he availability of temptations is large, and support from the surrounding environment is limited;* and (2) *n* *orms and demands set the agenda* that illuminates undermining factors concerning the adolescents’ healthy habits with regard to P.A. and food (Table 3). The categories are described and illustrated below by quotes that are characteristic for each category, using fictitious names, by illustrating if it is a boy (b) or a girl (g) and by indicating from which focus-group interview (e.g., FG1) the quote is retrieved. Moreover, each category begins by illuminating the adolescents’ own voices; this is followed by the findings’ relationships to previous research, and, when suitable, the findings are then interpreted through the theoretical frameworks.

**Table 3.** Research questions in relation to the main findings and its categories and themes.

Research question		Main findings	Category	Theme
What do the adolescents express as undermining factors in relation to healthy habits with regard to PA and food?				
		The adolescents expressed a profound awareness of and magnitude of screen-based activities (e.g., smartphone and tablet) as undermining their healthy habits with regard to PA and food.	Screen-based activities	The availability of temptations is large, and support from the surroundings is limited.
		The girls stated that when they were feeling sad or depressed, they tend to eat more sweet and high-fat foods, which undermined their healthy food habits.	Emotional eating	
		The adolescents conveyed that lack of time and inclement weather undermined their PA. Moreover, having a preference for unhealthy foods (e.g., fast food or candy) and a dislike towards the schools meals and healthy foods (e.g., fruit and vegetables) was mentioned as undermining the adolescents’ healthy food habits.	Priorities and preferences	
		Lack of social support from friends was stated as undermining the adolescents PA, while lack of parental support undermined both PA and healthy food habits.	Lack of social support	
		The adolescents articulated that they were dissatisfied with the lack of variety and options in the school cafeteria and that the availability of unhealthy foods in society at large made it difficult to eat healthy. Moreover, short breaks during the schooldays and long distances to sport clubs was mentioned as undermining the adolescents PA.	The environment and availability	
	How can the undermining factors expressed through the adolescents’ own voices be understood from a gender perspective?	The girls expressed that PA is not an activity for girls, but rather a masculine pursuit, that could be regarded as a symbolic boundary which undermined some of the girls PA. The girls also recognized a social boundary whereas sports are given an unequal distribution of resources.	Physical activity is mainly for boys	Norms and demands set the agenda
		The adolescents talked about how girls, but not boys, are expected to do household chores and only the girls mentioned homework as undermining the PA. Hence, there might exist symbolic boundaries that places greater expectations on girls to engage in household chores and greater demands on girls’ academic performance compared to that of boys.	Unequal demands concerning household chores and homework	
		Some of the girls stated that girls in general are concerned about their looks in relation to PA, and as such, they rather withdrawal from PA.	Focus on appearance	

### The availability of temptations is large, and support from the surrounding environment is limited

#### Screen-based activities

When the adolescents talked about what it is that prevents them from engaging in P.A, a common theme was different screen-based activities. The following thoughts were expressed when the adolescents were asked what it is that makes it difficult for them to be physically active:

Layla (g): *Mobile phones, iPad, computer, television.*

Naima (g): *Everything with technology.*

Layla (g): *Yes, exactly*. (FG2)

The adolescents expressed that, if they did not have access to smartphones and tablets, they would probably be healthier and more engaged in P.A, which aligns with the findings of Martins and colleagues (2015). Furthermore, on the one hand, some adolescents stated that it is more fun to interact and play games with their friends via smartphones, tablets, and computers than it is to be physically active. Others articulated that it is quite boring to use smartphones, tablets, and computers. They do so, however, because they have nothing else to do, and since their friends do it, it becomes a way to interact with them. In addition, the adolescents mentioned that video games, computer games, and smartphones are addictive to them, and that they sometimes lose track of time when they engage in these screen-based activities. When the adolescents lost track of time, they also mentioned that they sometimes forgot to eat, as illustrated below:

Anna (g): *Sometimes when I have food on the table and I’m using my phone, I completely forget the food. I’m using my phone, browsing.*

Yesenia (g): *And the food gets cold, and then you don’t want to eat it anymore*. (FG1)

Additionally, the adolescents stated that when playing video and computer games, they typically consumed unhealthy foods such as chips and soda. In a similar vein, screen-time has previously been associated with a high intake of, for example, energy-dense snacks and energy-dense drinks (Pearson & Biddle, 2011). Moreover, the adolescents stated that it is hard to stop playing video and computer games because they play them every day, and they are always available to them, unlike P.A, which is something they engage in only occasionally. The way in which the adolescents talked about screen-based activities indicates that these serve as a major barrier for them to engage in P.A. and to eat regularly. Moreover, the adolescents illuminated great awareness of screen-based activities undermining their P.A. and healthy food habits. As such, professionals (e.g., health pedagogies, teachers, and school nurses) working with adolescents should consider strategies for reducing adolescents’ screen-time activities and/or ways of utilizing smart devices (e.g., smartphones, tablets) in order to support the adolescents’ P.A. and healthy food habits.

#### Emotional eating

The girls in particular talked about how they tend to eat more sweet and high-fat foods (e.g., cookies, candy, potato chips) when they are feeling depressed, sad, or bored. The girls spoke about using food to “remove the sadness” and seeking comfort in food when no one else was there to comfort them, as illustrated below:

Yesenia (g): *If something has happened to you, and you really want it to go away. I eat, I comfort eat, and I listen to music.*

Amira (g): *Or if you are depressed. It feels like the food is there for you when no one else is around*. (FG1)

The adolescent girls’ stories about using food as a coping strategy illuminate what has been shown in research (Macht, 2008), namely that eating as a response to negative emotions and mood may result in the increased intake of sweet and high-fat foods.

#### Priorities and preferences

The adolescents talked about how lack of time may prevent them from engaging in P.A, which was illustrated by one of the girls as follows:

Maritza (g): *I used to dance at a dance club before. And I also took dance lessons. I think it was last year. But then I quit. I did not have the time for it*. (FG1)

Similarly, lack of time as a barrier to P.A. was identified by Martins et al. (2015). It has been argued, however, that the perception of lack of time is primarily an excuse to avoid P.A, and individuals citing lack of time as a barrier to P.A. may rather prioritize other leisure-time activities (Anshel, 2006). Moreover, the adolescents cited inclement weather as a barrier to P.A, which Anshel (2006) also highlights as a common barrier. Inclement weather could also be viewed as an excuse to avoid P.A, and that it might be easier for the adolescents to prioritize other activities when the weather is bad. In relation to eating healthy food, the adolescents spoke about how their taste preferences guided their food choices. Some of the adolescents stated that they did not appreciate the taste of some of the foods that they themselves defined as healthy, such as fruits and vegetables, while other adolescents mentioned that they had a preference for foods that they defined as unhealthy, such as fast foods and candy. Hence, taste preferences could be seen as an undermining factor for healthy food habits, which is similar to what Krølner et al. (2011) highlighted. In a similar vein, the adolescents mentioned a dislike for school meals, whereas the main concern was issues with taste and texture concerning the food that was served (e.g., the food tasted bad or was undercooked or overcooked; cf. Rawlins et al., 2013). Furthermore, some of the adolescents revealed that price was an important aspect of their food choices. When the adolescents were buying food, and there were many food items from which to choose, they stated that they usually bought the least expensive foods. Although not specifically mentioned by the adolescents, this may relate to what Krølner et al. (2011) underlined, namely that adolescents feel like they get more value for their money if they buy unhealthy options (e.g., candy, chips) compared to buying healthy options (e.g., fruits, vegetables; Krølner et al., 2011). With regard to taste preferences, professionals could encourage adolescents to challenge their taste preferences through sensory explorations, by engaging with foods with all their senses, which previously has been utilized to facilitate children and adolescents’ fruit and vegetable consumption (Dazeley, Houston-Price, & Hill, 2012).

#### Lack of social support

The adolescents expressed that if their friends were preoccupied with other commitments or simply did not feel like engaging in P.A, this served as a barrier for them to be physically active. Moreover, the adolescents declared that instead of nagging and being told what to do and what not to do in relation to P.A, they wished that their parents supported them and engaged in P.A. together with them. Additionally, the adolescents mentioned that lack of financial support from their parents makes it difficult for them to engage in organized P.A, a situation that, according to Martins et al. (2015), may be specific for adolescents from low socioeconomic areas. The lack of parental support was also mentioned by the adolescents in relation to healthy food habits. For example, they said that they lacked reminders about when to eat when their parents were absent from home; in addition, when their parents did not cook dinner for them and instead gave them money to buy food, they ordered unhealthy alternatives. This was illustrated in one of the focus groups as follows:

Interviewer: *Is there something that could be done so that it would become easier to eat more healthy foods and to eat more regularly?*

Sara (g): *At my place, nobody is at home. My mother works, my little sister she goes to different activities or something like that. My older sister, she’s in high school. My brother, I don’t know, and my dad, I think he works or something like that. So no one’s at home, it’s just me. And my little sister sometimes. So sometimes, I would like to have reminders*. (FG1)

Furthermore, the adolescents talked about how family members sometimes commented on and criticized their intake of energy-dense foods (e.g., candy, ice cream). These comments, according to the adolescents, were perceived as criticism and had a reverse effect on their intake of energy-dense food. The adolescents’ stories of how lack of social support from friends and family undermines their healthy habits with regard to P.A. and food are similar to what has been highlighted in previous studies (e.g., Jenkins & Horner, 2005; Martins et al., 2015). In relation to this, professionals working with adolescents could explore the adolescents’ sources of social support and encourage them to seek social support from family members and friends, as a way of supporting their healthy habits with regard to P.A. and food. Moreover, the adolescents stated that, when eating with friends, they ate mostly fast food such as McDonald’s, which was considered a hindrance for healthy food habits (cf. Shepherd et al., 2005).

#### The environment and availability

In relation to the school environment, some of the adolescents stated that they hardly ever ate in the school cafeteria (in Sweden, all elementary school students are provided with free school lunches), while others said that they ate there most of the time. The adolescents expressed that they were dissatisfied with the lack of variety and options for foods, as they stated that the same foods, more or less, were served each week and that only one dish was served each day. The adolescents also conveyed that the environment in the school cafeteria is noisy and unsanitary, which was illustrated in one of the focus groups as follows:

Interviewer: *How is it to eat there [at the school cafeteria]?*

Samir (b): *It is bad. There are many who don’t clean up after themselves.*

Johan (b): *[Other pupils] always scream.*

Samir (b): *[Other pupils] throw food.*

Yusuf (b): *It is chaos.*

Interviewer: *In what way? Tell us.*

Yusuf (b): *Like he said. No one cleans up after themselves, they scream and everything all the time. Plates are usually left on the tables*. (FG6)

Furthermore, the adolescents mentioned that it is difficult to eat healthy foods because of the availability of unhealthy foods (e.g., fast food) in society at large, which is similar to what adolescents in the study by Shepherd et al. (2005) stated. The adolescents also stated that there are several kiosks and pizzerias near the school, offering easy access to junk food and sweets. Moreover, short breaks during the schooldays and long distances to sports clubs, factors related to those found previously (e.g., Martins et al., 2015), were cited as barriers to P.A. by the adolescents. In other studies with adolescents from low S.E.S. areas, safety concerns in their neighbourhoods have been identified as barriers to P.A. (e.g., Rawlins et al., 2013), although this factor was not mentioned by the adolescents in this study. Thus, it is important for professionals to be aware of environmental factors influencing the adolescents’ P.A. and dietary habits (e.g., access to P.A. and the availability of unhealthy foods) and to identify strategies for the adolescents to manage these factors.

### Norms and demands set the agenda

#### P.A. is mainly for boys

Some of the girls expressed that they do not engage in P.A. because it is not an activity for girls. Moreover, the girls stated that girls should not engage in sports, since “they cannot” and because they are afraid of embarrassing themselves due to lack of skills and competencies. In a similar vein, the girls highlighted that boys have more skills and competencies in relation to P.A. Furthermore, the girls mentioned that the options for physical activities are less for girls compared to boys. This was illustrated by the following discussion:

Mina (f): *There is more for boys; soccer, for example, that is a sport for boys, I think.*

Interviewer: *Why do you think that soccer is considered a sport for boys?*

Kayla (f): *Because it’s usually not a lot of girls who train.*

Maria (f): *You see more guys playing soccer on TV*. (FG10)

Moreover, some of the girls mentioned that during school breaks and after school hours, several of the boys gather at a nearby soccer field to play soccer. The girls, however, were allowed to participate only occasionally, and the boys usually decided who could play and who could not. The adolescent girls’ statements, which describe P.A. as a masculine pursuit, could be regarded as a symbolic boundary (Lamont & Molnár, 2002) that undermines some of the girls’ P.A. in this study. Similarly, Spencer and colleagues (2015) showed that adolescent girls acknowledge a male dominance in sports and that sports is not for girls. Moreover, Spencer and colleagues (2015) argues that there is a great complexity surrounding girl’s relationship with P.A. On the one hand, girls may feel pressured to appear and act feminine, which limits their ability to behave outside these norms. On the other hand, some girls may challenge these norms, but, in doing so, they also risk being perceived as too masculine (Spencer et al., 2015). In addition, the girls in this study recognized a social boundary in that sports are given an unequal distribution of resources (Lamont & Molnár, 2002); in this case, women’s sports receive less attention and less media coverage than men’s sports (cf. Packer et al., 2015).

#### Unequal demands concerning household chores and homework

The adolescents talked about how girls are expected to do household chores at home that occupy their time and may prevent them from being physically active, as exemplified in one of the focus groups:

Jazmin (g): *It may well be that one has to do something at home, for example… I always do the dishes at home.*

Interviewer: *Do you need to help out a lot at home?*

Mona (g): *Yes.*

Sanaa (g): *Yes.*

Alma (g): *I do the dishes a lot, always*. (FG3)

The adolescents also expressed that girls, in general, help more in the household, while boys are not expected to bring the same effort into the household and thus have more time to engage in P.A. This is consistent with previous research that highlights that girls’ housework may hinder them from engaging in P.A. (Humbert et al., 2008). In relation to doing household chores, the girls also spoke about caring for their siblings and feeding them, which sometimes causes them to forget to eat. This might, however, be specific for adolescents from low S.E.S. families since previous research has stressed that girls (Humbert et al., 2008) and boys (Dagkas & Stathi, 2007) from such families mention family obligations as a barrier to P.A, while adolescents from high S.E.S. families do not (Dagkas & Stathi, 2007; Humbert et al., 2008). Furthermore, only the girls in this study spoke about school homework and how it prevents them from going outside on weekdays, and as a result, it is difficult to prioritize P.A. (cf. Humbert et al., 2008). In relation to household chores, the adolescent girls’ statements reveal that a symbolic boundary might exist (Lamont & Molnár, 2002) that separates girls from boys and places greater expectations on girls compared to boys to engage in household chores. Similarly, a symbolic boundary might exist (Lamont & Molnár, 2002) that places greater demands on adolescent girls’ academic performance compared to that of boys.

#### Focus on appearance

When some of the girls spoke about how they could help other girls to become physically active, they stated that, in general, girls are concerned about their looks in relation to P.A. More specifically, they talked about girls not wanting to ruin their makeup and hair and not wanting to get sweaty, and as such, they prefer to withdraw from P.A, as illustrated below:

Interviewer: *If you would try to help other girls to become more physically active, how would you do it?*

Sara (g): *I don’t think they would care. They only care about makeup, if their makeup would go away, if they get sweaty and their mascara goes away* (FG2).

The adolescent girls’ concerns about their physical appearance and ruined makeup in relation to P.A. are in accordance with earlier studies (e.g., Spencer et al., 2015). Hence, it is possible that there may be a symbolic boundary present (Lamont & Molnár, 2002), which pressure some girls to maintain a good appearance while being physically active. Or, rather, the appearance fixation can undermine girls’ willingness to engage in P.A. since it may challenge their femininity, which is similar to what Spencer et al. (2015) emphasized in their review. Related to this, and the symbolic boundaries recognized in the two previous categories, professionals working with adolescents should demonstrate an awareness concerning gender norms and ideals related to P.A. and healthy food habits and inspire adolescents to challenge these gender norms and ideals.

From a theoretical perspective, this study has provided a deeper understanding of how gender boundaries (both symbolic and social) undermine some adolescent girls’ opportunities for healthy habits with regard to P.A. and food. This has been accomplished through listening to the voices of adolescents’ from a multicultural community with low S.E.S, adolescents that previously has been overlooked.

The majority of previous studies with children and/or adolescents in multicultural and low S.E.S. communities has either studied P.A. or healthy eating in isolation from one another (e.g., McEvoy et al., 2016; Taverno Ross & Francis, 2016; Tiedje et al., 2014), there are, however, some exceptions (e.g., Rawlins et al., 2013). The adolescents in this study did highlight factors that undermine their P.A. and healthy food habits that had been unnoticed in these previous studies. Such as, the magnitude of screen-based activities as undermining their healthy habits with regard to P.A. and food, and the existence of symbolic and social gender boundaries undermining the girls P.A. and healthy food habits. Nonetheless, some barriers to P.A. and healthy eating identified in previous studies were not emphasized by the adolescents in this study (see Rawlins et al., 2013; Tiedje et al., 2014; for example). For instance, Rawlins and colleagues (2013) recognized safety concerns in the children’s neighbourhoods as a barrier to P.A, and Tiedje and colleagues (2014) acknowledged cultural foods and traditions as a barrier to eating healthy. Overall, however, the main findings of the present study seem to be in line with those of other scholars.

## Method discussion

In order to increase the trustworthiness of our findings, we followed the recommendations proposed by Graneheim and Lundman (2004), as illustrated below. To ensure credibility, we took the following three measures: (1) When selecting the sample, we invited all seventh graders in the targeted school to participate in the study, and all agreed; however, one of the adolescents was absent during the focus-group interview; (2) Focus-group interviews are an effective method for exploring how people think and speak about certain subjects and to recognize the world from the participants’ perspectives (Dahlin Ivanoff & Hultberg, 2006); the choice of this method can thus be considered appropriate for the purpose of the study; (3) Furthermore, the meaning units selected were neither too broad nor too narrow (see examples in Table 2), and to ensure that no relevant data were excluded or irrelevant data included, categories and themes was regularly discussed among the co-authors during the analysis process, as recommended by Graneheim and Lundman (2004). At the stage of analysis when all the meaning units had been condensed and coded, they were reviewed by the co-authors, and similarities and differences were discussed until consensus was reached. This was done to enhance the dependability of the study findings (cf. Graneheim & Lundman, 2004). Moreover, in order to assist the readers to draw conclusions regarding the transferability of the study findings, we have attempted to present a clear and distinct description of the context as well as the data collection and analytic procedures. A limitation, however, is that we did not gather any additional background information, than age and gender, about the participants.

It was our intention to conduct traditional focus-group interviews as specified in the literature (e.g., Dahlin Ivanoff & Hultberg, 2006). However, we experienced that the adolescents in some of the focus groups were, to some extent, only answering our questions. One explanation for this may relate to the power relations embedded in the very nature of conducting research interviews (e.g., Greene & Hohan, 2005; Vähäsantanen & Saarinen, 2013). More specifically, during each focus-group interview, there were three adults present which probably contributed to a great power imbalance. Furthermore, we experienced differences within and between the focus groups concerning the adolescents’ ability to express themselves. Overall, between the groups it is our appraisal that the girls were more articulate than the boys; this may be related to adolescent girls’ emotional and cognitive functioning, in general, maturing earlier than in adolescent boys (Lim, Han, Uhlhaas, & Kaiser, 2015). It may also be the case that the girls had reflected more upon the topics discussed compared to the boys. Within the groups, we also experienced that there were usually two or three adolescents who were more articulate than their peers. To some extent, this can be explained through language barriers, as the school receives a number of newly arrived immigrants, and there were a few adolescents in the focus groups who could not speak Swedish fluently. Hence, a limitation is that we did not have an interpreter present for those adolescent who did not speak Swedish fluently. Lastly, another limitation with the study was that the main moderators in the focus-groups was neither trained nor experienced (other than the pilot focus-groups), which is important for the success of the focus group process (Roller & Lavrakas, 2015). There was, however, a senior researcher, with experience of conducting focus-group interviews, present during each focus-group.

## Conclusion

In conclusion, according to the perspectives of the adolescents in this study, there are several factors undermining their possibility of establishing healthy habits with regard to P.A. and food at an individual level (e.g., screen-based activities), a social level (e.g., lack of social support), an environmental level (e.g., the school environment), and a societal level (e.g., gender norms). Based on the adolescents’ voices the factors that seem to be specific for them were a profound awareness of and magnitude of screen-based activities as undermining healthy habits with regard to P.A. and food; these are not comparable, or sometimes not even highlighted, in previous studies (cf. Bragg, Tucker, Kaye, & Desmond, 2009; Tiedje et al., 2014). The adolescents’ stories could also be interpreted as the existence of symbolic gender boundaries that undermine the girls’ P.A. and healthy food habits. That is, on the one hand, P.A. was mainly considered an activity for boys, which undermined the girls’ willingness to engage in P.A. On the other hand, the girls highlighted that they are expected to engage in household chores, e.g., cooking, cleaning, and caring for younger siblings, which undermined their possibilities for P.A. and healthy food habits. The boys, by contrast, were not expected to bring the same efforts to the households. Moreover, the adolescents highlighted that the school cafeteria was a noisy and unsanitary environment and, as such, served as a barrier to consuming lunch at school.

The findings of the present study may be used to develop actions aiming at promoting P.A. and healthy food habits, with the potential for improving the adolescents’ health and well-being. When designing and implementing forthcoming actions that aim to facilitate P.A. and healthy food habits among adolescents, we suggest that it is important to provide support at multiple levels. Moreover, seeing how difficult it is for adolescents to establish healthy habits with regard to P.A. and food, there is also a need for change at a societal level (e.g., a wider range of free and easily accessible facilities/recreational areas that encourages P.A.) and for political decisions (e.g., taxes on sugar sweetened beverages and/or reduced value-added taxes on healthy food options) that are supportive of the adolescents’ healthy habits.

To develop actions aiming at promoting healthy habits with regard to P.A. and food, it is not only important to have an understanding of undermining factors. As such, for future directions, it might be of interest to gain insight about factors that can potentially facilitate healthy habits with regard to P.A. and food among adolescents from multicultural communities with low S.E.S.
